# Analysis of laboratory blood parameter results for patients diagnosed with COVID‐19, from all ethnic group populations: A single centre study

**DOI:** 10.1111/ijlh.13538

**Published:** 2021-05-03

**Authors:** Mandeep Marwah, Sukhjinder Marwah, Andrew Blann, Hana Morrissey, Patrick Ball, Farooq A. Wandroo

**Affiliations:** ^1^ Aston University Birmingham UK; ^2^ Department of Haematology Sandwell and West Birmingham Hospitals, NHS Trust West Bromwich UK; ^3^ The Institute of Biomedical Science London UK; ^4^ University of Wolverhampton Wolverhampton UK

**Keywords:** biochemistry, COVID‐19, ethnic groups, haematology, mortality indicators

## Abstract

**Introduction:**

Although factors such as age, sex, diabetes, obesity and changes in certain laboratory investigations are important prognostic factors in COVID‐19 infection, these may not apply to all ethnic/racial groups. We hypothesized differences in routine biochemistry and haematology indices in Caucasian and a combined group of Black, Asian and Minority Ethnic (BAME) patients who tested positive for COVID‐19 who died, compared to survivors.

**Methods:**

We tested our hypothesis in 445 patients (229 Caucasian, 216 BAME) admitted to secondary care with proven COVID‐19 infection, in whom standard routine laboratory indices were collected on admission.

**Results:**

After 28 weeks, 190 (42.7%) had died within 28 days of COVID diagnosis (97 Caucasians [42.4%], 93 BAMEs [43.1%], *P* = .923). A general linear model analysis found the ethnicity interaction with mortality to be significant for fibrinogen, ferritin and HbA_1_c (after controlling for age). In a multivariate analysis, a neutrophil/lymphocyte ratio > 7.4 and a urea/albumin ratio > 0.28 increased the odds of death for both the Caucasian and the BAME group. Additional factors increasing the odds ratio in the BAME group included age >60 years and being diabetic.

**Conclusion:**

Neutrophil/lymphocyte ratio and urea/albumin ratio are simple metrics that predict death to aid clinicians in determining the prognosis of COVID‐19 and help provide early intensive intervention to reduce mortality. In the BAME groups, intensive monitoring even at younger age and those with diabetes may also help reduce COVID‐19 associated mortality.

## 1 INTRODUCTION

The severe acute respiratory syndrome coronavirus 2 (SARS‐COV‐2) is an ribonucleic acid (RNA) virus that spreads rapidly via droplets, aerosols and contaminated surfaces.^1^ Clinical Coronavirus disease 2019 (COVID‐19) presentation ranges from asymptomatic, through to mild influenza‐like symptoms to multiple organ failure and death.^2^, ^3^, ^4^ The reported mortality worldwide ranges from 8.1% to 33% in hospitalized patients.^2^, ^5^, ^6^ Gender, age, comorbidities including diabetes, hypertension and obesity, changes in laboratory investigations including haematological and biochemical characteristics in patients with COVID‐19 are emerging as important prognostic factors.^2^, ^7^ Many of these laboratory indices, including a decreased platelet count and increased prothrombin time, ferritin, lactate dehydrogenase, D‐dimer and neutrophil/lymphocyte ratio, have also been linked to high mortality in a South Asian population.^8^

In the United Kingdom (UK), cases within patients from Black (African and Afro‐Caribbean), Asian and Minority Ethnic (BAME) groups have a worse prognosis than that seen in Caucasians.^9^, ^10^, ^11^ Perkin et al^12^ reported deaths in patients from the BAME communities in the COVID‐19 group during the pandemic period to be significantly increased (*P* = .02). The increase was reported to be independent of comorbidities, sex, age or socioeconomic deprivation. Major risk factors for mortality were male sex, diabetes mellitus, multiple comorbidities and being from the BAME communities. Bannaga et al^11^ found BAME patients were more likely to require intensive care unit (ICU) admission (*P* = .008).

Many hospitals across the world have been collecting data prospectively as patients with COVID‐19 first presented, looking for patterns in clinical findings and routine laboratory markers that may predict risk of a poor health outcome in a variety of patient groups. Collected during an emerging pandemic, many of these series have limitations, but in addition to learning from their individual outcomes, publication offers the possibility of future data pooling and meta‐analysis to increase the reliability of the findings. Early in the pandemic, the importance of coagulation and inflammatory markers was noted.^13^ Bannaga et al^11^ noted increased mortality from COVID‐19 was associated with age >65 years (*P* = .026), heart disease (*P* = .009) and elevated C‐reactive protein (CRP) (*P* = .011), raised neutrophils (*P* = .047) and altered neutrophil/lymphocyte ratio (*P* = .028). Taj et al^14^ concluded that leucocytosis, neutrophilia, lymphopenia, elevated neutrophil/lymphocyte ratio, activated partial thromboplastin time (APTT), fibrinogen and fibrin degradation product (D‐dimer), lactate dehydrogenase (LDH), serum ferritin and CRP are associated with severity of COVID‐19 disease.

As 50% of the population in our catchment area are from ethnic minorities,^15^ the region has been seeking to identify the reasons for the difference in outcomes in BAME patients. We hypothesized that certain routine haematology and biochemistry indices may be altered in those Caucasians and BAME patients who subsequently died within 28 days of a positive COVID‐19 test and that pattern identification would facilitate prediction of those at high risk of severe disease and rapid deterioration.

## 2 MATERIALS AND METHODS

### 2.1 Study design and participants

This retrospective cohort study included all admitted patients testing positive for COVID‐19 from a single centre National Health Trust in the UK between 3rd February 2020 and the 4th September 2020. Data from 445 patients (>18 years) were included in the analysis, 252 were males and 193 were females. Of the 255 patients that survived, 132 were Caucasian and 123 were from BAME group. Of these, coding showed that 23 were Black, 56 were Asian, 8 were mixed race and 36 were only coded as BAME without a subgroup code. Of the 190 patients that died, 97 were Caucasian, and 93 were from the BAME group (Black 32, Asian 41, mixed race 3, not coded 17). Patients were classified as Caucasian or BAME, and as survivors or fatalities. Furthermore, 10% (46 out of 445) of patients were admitted to intensive care unit (ICU) of which 70% (32 out of 46) patients were from BAME group. This study was granted ethical approval by the Integrated Research Approval System (IRAS) and sponsored by research and development committee of the Trust (IRAS number 289571). All patients were treated using the local trust and the NHS protocols irrespective of their ethnicity. All patient results were collected within 3 days of COVID‐19 diagnosis.

### 2.2 Laboratory procedures

Patients were identified as COVID‐19 positive by reverse transcriptase polymerase chain reaction (RT‐PCR) from throat/nose swabs on a ROCHE COBAS™ analyser (Roche Ltd.). Nasopharyngeal or oropharyngeal samples were collected from patients for the detection of SARS‐CoV‐2 RNA. The Xpert^®^ Xpress SARS‐CoV‐2 (Cepheid Ltd.) real‐time RT‐PCR assay was performed to achieve qualitative detection of SARS‐CoV‐2 RNA.

A Sysmex™‐XN (Sysmex LTD.) automated haematology analyser was used for routine full blood count analysis. Instrumentation Laboratory ACL350™ and ACL550™ analysers (Werfan Ltd.) were used to measure the prothrombin time, from which the internal normalized ratio (INR) was calculated, the partial prothrombin time (PTT) and the D‐dimer level. An Abbot Architect™ analyser was used to determine quantitative biochemical analysis including urea and electrolytes, albumin, alkaline phosphatase (ALP), alanine aminotransferase (ALT), creatinine, LDH, troponin, CRP and ferritin.

### 2.3 Statistical analysis

Continuous variables were presented as either median (interquartile range) or mean/(±standard deviation) depending on distribution (distribution of normality was checked by D’Agostino Pearson's normality test). Continuous variables were analysed using a Kruskal‐Wallis test with multiple comparisons among groups analysed using the Bonferroni adjustment method test or a one‐way ANOVA analysis with a post hoc Tukey's test where appropriate. Furthermore, a Mann‐Whitney *U* test was used to determine any differences between the Black and Asian population within the BAME group. To determine interaction between ethnicity and morbidity on the dependent variable, a general linear model was used adjusting for age. A two‐sided test *P* < .05 was considered statistically significant. Sample size varied because of missing data as detailed in the tables. Data were subject to univariate analyses: those with a significance <0.05 were subsequently analysed by multivariate methods.

To explore the risk factors associated with mortality for Caucasian and BAME patients, multivariate logistic regression models were used. Considering the total number of deaths per group (97 and 93 within the Caucasian and BAME groups, respectively) in this study and to avoid over fitting in the model, four variables were chosen for multivariable analysis for both the Caucasian and BAME group on the basis of a univariate analysis. Variables were excluded from the univariate analysis if the number of events was too small to calculate the odds ratio and if the receiver operator characteristic (ROC) area under the curve was <0.6. For the ratios proposed, the ROC was used to evaluate optimal thresholds for individual biomarkers. A multivariate logistic regression model was then fitted, to estimate the effect of indicators on inpatient mortality for critically ill patients. Based on the univariate analysis for the Caucasian group, diabetes, neutrophil/lymphocyte ratio, urea/albumin ratio and the (ALP × ALT)/albumin index showed the highest discriminatory power and were chosen as variables for our multivariate logistic regression model. Based on the univariate analysis for the BAME groups, age, diabetes, neutrophil/lymphocyte ratio and urea/albumin ratio were selected as variables for our multivariate logistic regression model. A two‐sided *α *< .05 was considered statistically significant. All statistical analyses were performed using SPSS™ software (version 26).

## 3 RESULTS

Table 1 shows clinical and demographics of the subjects, limited to those were captured on admission. Survivors, in both groups, were younger than those who died. In the study sample, diabetes was not linked to Caucasian deaths but was strongly linked to BAME deaths. The majority of patients where body mass index (BMI) was possible to be calculated were overweight (151 out of 233 patients), and nearly half were diagnosed with diabetes (199 out of 445 patients). Hypertensive presentation and a raised heart rate (HR > 100) on admission were not prevalent; however, an increased respiratory rate (RR) was observed (>20 breaths per minutes, in 175 out of 411 patients). Further, a peripheral venous oxygen saturation (SpO_2_) < 94 was prevalent in approximately 25% of all patients. Furthermore, of the Caucasian men, 44% died, of the Caucasian women, 40% died whereas of the BAME group men, 48% died, and of the BAME women, 37% died.

**TABLE 1:** Demographic and clinical observations findings of patients on admission

	*P* value	Caucasian (C)		*P* value	BAME (B)		*P* value
	Overall	Survivors (S)	Fatalities (F)	CS vs CF	Survivors (S)	Fatalities (F)	BS vs BF
Age (y)	<0.0001	78 [68‐87]	81 [73‐87.5]	.98	56 [41.75‐73.25]	79 [67‐85]	<.0001
N		132	97		123	93	
Gender							
Male	.613	73	57		64	58	
Female	.661	59	40		59	35	
Body mass index	.103	25.5 [22.8‐31.2]	27.2 [23.4‐31.6]	.107	28.3 [25.5‐33.9]	26.7 [23.2‐31.7]	.999
n		75	48		72	38	
Diabetes	.151	46 (36%)	39 (40%)	.84	49 (42%)	65 (68%)	<.001
n		127	97		118	93	
Hypertension >140/90 in mm Hg	.208	19/116 (16%)	18/96 (19%)		20/107 (19%)	9/93 (10%)	
n		116	96		107	91	
Respiratory Rate (breaths/min)	.012	19.5 [18‐22]	20 [18‐24]	.808	20 [18‐24]	23 [18‐27]	.999
n		116	96		108	91	
Heart rate (beats/min)	.832	83 [69‐99]	88 [77‐101]	.182	90 [80‐101]	89 [79‐106]	.999
n		116	96		108	91	
SpO_2_ (%)	.590	96 [94‐97]	96 [93‐97]	.999	96 [94‐98]	96 [93‐98]	.999
n		116	96		108	91	

Note

Data are median (IQR), n (%), or n/N (%). *P* values were calculated by a Kruskal‐Wallis test with a post hoc Dunn's multiple comparison test or Fisher's exact test, as appropriate.

Table 2 shows haematology data. Differences in the haemoglobin (Hb) across men and women, the mean corpuscular volume (MCV), white blood cell count (WBC), neutrophil, lymphocyte, monocyte and eosinophil count, INR, D‐dimer and HbA_1c_ concentrations (*P* < .05, for all parameters) were observed across Caucasian and BAME between survivors and fatalities. The median MCV value was higher in fatalities compared with survivors. However, MCV values were significantly lower in BAME survivors and fatalities data than that seen in Caucasians (overall *P* ≤ .0001). This study demonstrated significantly higher WBC and neutrophil count as well as a lower lymphocyte count in the Caucasian fatality and survivors group, compared with BAME fatality and survivors groups (overall *P* = .004, *P* ≤ .001 and *P* ≤ .0001, respectively). Lymphopenia was significant in the fatality groups compared to their respective survivors (*P* = .021 and *P* ≤ .0001 in the Caucasian and BAME group, respectively). Finally, the generalized linear model (GLM) analysis (Table S1) found the ethnicity factor (BAME group) to be significant for monocyte count (*P* = .007).

**TABLE 2:** Haematological findings of COVID‐19 positive patients on admission

	*P* value	Caucasian (C)		*P* value	BAME (B)		*P* value
	Over all	Survivors (S)	Fatalities (F)	CS vs CF	Survivors (S)	Fatalities (F)	BS vs BF
Hb, g/L							
Male	.023	132 (112‐151)	129 (102‐157)	.999	130 (109‐152)	120 (98‐142)	.066
<125		27/73 (40%)	23/56 (41%)		23/64 (36%)	33/58 (57%)	
Female	.044	123 (103‐143)	117 (92‐142)	.607	118 (101‐135)	109 (83‐135)	.248
<115		23/59 (40%)	18/40 (45%)		22/43 (51%)	20/32 (63%)	
MCV (fL)	<.0001	90.9 [88.1‐95.4]	94.3 [89.85‐98.35]	.022	86.2 [81.2‐90.6]	89.2 [84.6‐94.23]	.011
WBC × 10^9^/L	.004	8.5 [6.2‐11.3]	9.55 [6.5‐12.5]	.991	7.3 [5.65‐9.45]	8.85 [6.96‐11.80]	.019
>11		36/132 (27%)	35/96 (36%)		25/122 (20%)	26/90 (29%)	
Neutrophil count × 10^9^/L	<.0001	6.78 [4.41‐9.7]	7.92 [5.14‐10.71]	.168	5.33 [3.85‐7.44]	7.17 [5.55‐10.38]	.001
>7.5		49/132 (37%)	50/96 (52%)		30/122(25%)	41/90 (46%)	
Lymphocyte count × 10^9^/L	<.0001	0.95 [0.6‐1.35]	0.725 [0.53‐0.99]	.021	1.15 [0.81‐1.53]	0.77 [0.55‐1.23]	<.0001
<1		70/132 (53%)			46/132 (35%)	56/90 (62%)	
Monocyte count × 10^9^/L	.008	0.58 [0.37‐0.82]	0.58 [0.29‐0.82]	.999	0.46 [0.31‐0.63]	0.46 [0.26‐0.69]	.999
<0.2		6/132 (5%)			13/132 (10%)	17/90 (19%)	
>0.8		33/132 (25%)			20/132 (15%)	12/ 90 (13%)	
Eosinophil count × 10^9^/L	.005	0.02 [0.005‐0.08]	0.01 [0.005‐0.03]	.005	0.01 [0.005‐0.07]	0.005 [0.005‐0.053]	.999
Platelet count × 10^9^/L	.136	242 [187‐303]	218 [154‐280]	.999	231 [182.5‐320]	227.5 [174.8‐295.5]	.999
<150		13/132 (10%)	20/95 (21%)		16/132 (12%)	12/90 (13%)	
INR	.002	1.18 [1.1‐1.29]	1.275 [1.12‐1.48]	.147	1.15 [1.07‐1.25]	1.19 [1.12‐1.35]	.177
>1.2		42/95 (44%)	43/72 (60%)		31/89 (35%)	32/72 (44%)	
PTT	.072	1.01 [0.91‐1.07]	1.065 [0.99‐1.31]		0.89 [0.87‐1.03]	1.055 [0.9425‐1.495]	
>1.2			4/10 (40%)			4/12 (33%)	
D‐dimer (FEU µg/ml)	.004	1.07 [0.89‐15.25]	2.36 [1.05‐12.35]	.999	1.63 [0.61‐1.96]	7.52 [2.13‐31.93]	.003
>0.5		11/11 (100%)	12/13 (92%)		13/15 (87%)	24/25 (96%)	
Fibrinogen (g/L)	.118	2.2 [1.4‐3.0]	6.6 [4.1‐9.8]		7.2 [5.2‐8.9]	8.5 [5.25‐10]	
>4		1/2 (50%)	8/11 (72%)		6/7 (86%)	18 (86%)	
Ferritin (µg/L)	.459	649.5 [225.8‐1126]	647.5 [231.8‐1526]	.999	556.5 [176‐1550]	686.0 [202.5‐2508]	.999
>300		50/72 (69%)	34/50 (68%)		42/66 (64%)	35/49 (71%)	
HbA_1c_ (mmol/mol)	<.0001	41 [38‐47]	42 [38‐49.5]	.999	43.5 [40‐55.5]	51 [44‐67]	.006
>42		46/112 (41%)	39/85 (46%)		49/88 (56%)	65/83 (78%)	

Note

Non‐normally and normally distributed continuous variables are presented as medians (interquartile ranges, IQR) and mean (standard deviation), respectively, as well as n/N (%). Continuous variables were analysed using a Kruskal‐Wallis test with a post hoc Dunn's multiple comparison test or a one‐way ANOVA with a post hoc Tukey's test where appropriate.

The median D‐dimer and fibrinogen levels of all four groups were above the reference range (0.5 µg fibrinogen equivalent units (FEU)/mL and 4 g/L, respectively) of the available patient data (Table 2). The D‐dimer levels were higher in those who died in both groups, but the relative increase was greater in the BAME group. Furthermore, the fibrinogen level was significantly higher in the Black fatality population compared with Asian fatalities (*P* < .03), (Table S2). The GLM analysis (Table S1) found the mortality and ethnicity factor interaction to be significant for fibrinogen with a possible significant effect of ethnicity on mortality (*P* = 024 and 0.25, respectively). The median HbA_1_c level was significantly different across the groups (*P* ≤ .0001), the BAME group having higher values (Table 2). The GLM analysis adjusted for age found the mortality (*P* = .007) with ethnicity (*P* ≤ .0001) factor to be significant for HbA_1_c; thus, there was a significant effect of ethnicity on mortality (Table S1).

Table 3 shows biochemistry results. Differences in the urea, sodium, potassium, albumin, creatinine, ALT, CRP, LDH and troponin‐I (*P* < .05) levels were observed across Caucasian and BAME survivors and fatalities. In the subgroup analysis, MCV, lymphocyte, eosinophil, urea, sodium, albumin, CRP, LDH and troponin‐I were significantly different in Caucasian survivors compared to Caucasian fatalities. Similarly, MCV, WBC, neutrophil, lymphocyte, D‐dimer, HbA_1c_, urea, sodium, potassium, albumin, creatinine, CRP and troponin‐I were significantly different in BAME survivors compared BAME fatalities. Serum urea (*P* ≤ .0001 for both Caucasian and BAME) and serum sodium (*P* = .027 and *P* ≤ .0001 for Caucasian and BAME, respectively) concentrations were significantly higher in the fatality groups, whereas serum albumin was significantly lower in both fatality groups when compared with each survivor group (*P* = .0002 and *P* ≤ .0001 for Caucasian and BAME groups, respectively). The potassium and creatinine levels in the BAME fatality group were significantly higher compared with both Caucasian groups (*P* = .003 and *P* ≤ .0001, respectively). The urea to albumin ratio was significantly different across the groups at *P* ≤ .0001 (Table 3). The GLM analysis (Table S1), adjusted for age, observed the mortality and ethnicity interaction was significant for this ratio (mortality *P* = .005 and ethnicity *P* ≤ .0001). Of the liver function tests, the ALT concentration in the Caucasian group was significantly lower compared with BAME group (*P* = .002). The (ALP × ALT)/albumin index was significantly different across the groups (*P* ≤ .0001).

**TABLE 3:** Biochemical findings of COVID‐19 positive patients on admission

	*P* value	Caucasian (C)		*P* value	BAME (B)		*P* value
	Over all	Survivors (S)	Fatalities (F)	CS vs CF	Survivors (S)	Fatalities (F)	BS vs BF
Urea (mmol/L)	<.0001	8.05 [5.325‐11.48]	11.15 [8.125‐19.2]	<.0001	6.1 [4.5‐10.6]	13.6 [8.85‐21.48]	<.0001
>7.8		65/129 (50%)	74/96 (77%)		42/121 (35%)	74/90 (82%)	
Sodium (mmol/L)	<.0001	137 [135‐141]	140 [137‐143.8]	.027	136 [133‐139]	140 [134‐143.3]	<.0001
<133		18/129 (14%)	8/96 (8%)		21/121 (17%)	12/90 (13%)	
>146		16/129 (12%)	15/96 (16%)		4/121 (3%)	18/90 (20%)	
Potassium (mmol/L)	.003	4.01 (3.36‐4.66)	4.07 (3.36‐4.77)	.999	4.14(3.56‐4.72)	4.45 (3.51‐5.38)	.021
<3.5		27/122 (22%)	18/89 (20%)		11/109 (10%)	8/84 (10%)	
Albumin (g/L)	<.0001	35.61 (30.40‐40.82)	32.30 (27.07‐37.53)	.0002	36.45 (30.61‐42.29)	31.99 (25.90‐38.1)	<.0001
<5		49/115 (43%)	61/91 (67%)		37/104 (36%)	54/87 (62%)	
Creatinine (µmol/L)	<.0001	89.5 [72‐118.8]	104.5 [75‐162]	.068	94 [72‐124]	132 [88.75‐239]	<.0001
>133		24/124 (19%)	35/96 (36%)		25/115 (22%)	44/90 (49%)	
ALP (U/L)	.195	89 [74‐118]	93 [74‐139]	.999	83.5 [65‐115.8]	87 [62.5‐116.5]	.999
>130		23/115 (20%)	25/91 (27%)		22/104 (21%)	19/85 (22%)	
ALT (U/L)	.002	25 [17‐38]	26 [17‐52]	.676	32 [21‐57]	34 [18.5‐58]	.999
>41		21/113 (19%)	30/91 (33%)		55/103 (53%)	34/85 (40%)	
Bilirubin (µmol)	.061	11 [8‐17]	12 [8‐18]	.999	10 [7‐17]	11 [7‐16]	.999
>21		17/114 (15%)	9/90 (10%)		5/104 (5%)	9/83 (11%)	
CRP (mg/L)	<.0001	98 [31‐168]	130 [70.75‐235.5]	.012	83 [40‐174]	157 [102‐246]	<.0001
>1		107/111 (96%)	92/92 (100%)		102/103 (99%)	85/86 (99%)	
LDH (U/L)	<.0001	310 [235‐405.5]	444 [278.5‐660]	.034	417 [304.3‐549.5]	486.5 [297.5‐698.5]	.999
>5		65/65 (100%)	43/45 (96%)		56/56 (100%)	40/42 (95%)	
Troponin‐I (ng/L)	<.0001	26.5 [13‐69.75]	71 [24.5‐201.5]	.052	11 [4‐31]	63 [29.5‐212]	<.0001
>3		60/60 (100%)	59/61 (97%)		51/67 (76%)	61/61 (100%)	

Note

Non‐normally and normally distributed continuous variables are presented as medians (interquartile ranges, IQR) and mean (standard deviation), respectively, as well as n/N (%). Continuous variables were analysed using a Kruskal‐Wallis test with a post hoc Dunn's multiple comparison test or a one‐way ANOVA with a post hoc Tukey's test where appropriate. For variables with overall *P* > .05, we did not perform group comparisons.

The median CRP, ferritin and LDH concentration of both Caucasian and BAME groups remained above the reference range (Tables 2 and 3). The troponin‐I level in both fatality groups was significantly higher compared with both survivor groups. Further, the LDH concentration in the Caucasian survivor group was significantly lower compared with both fatality groups. Both the mortality (*P* = .023) and ethnicity (*P* = .043) interaction was significant for ferritin; thus, there was a significant interaction/effect of ethnicity on mortality (Table S1). Further subgroup analysis into the BAME group revealed both the LDH (*P* = .006) and troponin‐l (*P* = .04) were significantly higher in Black fatality population (LDH 682 [521‐846] and troponin‐I 140 [60‐353]) compared with Asian fatalities (LDH [406/253‐626] and troponin [46/22‐166] (Table S2).

The neutrophil‐to‐lymphocyte ratios were significantly higher in both fatality groups compared to their respective survivors (*P* ≤ .0001, Caucasians at *P* = .006 and BAME at *P* ≤ .0001) (Table 4).

**TABLE 4:** Ratios are presented as medians (IQR)

	*P* value	Caucasian (C)		*P* value	BAME (B)		*P* value
	Overall	Survivors (S)	Fatalities (F)	CS vs CF	Survivors (S)	Fatalities (F)	BS vs BF
Neutrophil/lymphocyte	<.0001	7.0 (3.7‐13)	10.4 (6.3‐16)	.006	4.5 (3.2‐8.1)	9.4 (5.6‐17)	<.0001
Urea/Albumin	<.0001	0.22 (0.16‐0.35)	0.34 (0.29‐0.61)	<.0001	0.17 (0.11‐0.27)	0.40 (0.28‐0.68)	<.0001
(ALP × ALT)/albumin	<.0001	66 (36‐125)	46 (71‐177)	<.0001	80 (44‐186)	87 (47‐197)	<.0001

Note

Continuous variables were analysed using a Kruskal‐Wallis test with a post hoc Dunn's multiple comparison test.

In the univariate logistic regression analysis for the Caucasian group, a raised creatinine, CRP, neutrophil/lymphocyte, urea/albumin and (ALP × ALT)/albumin index, as well as increasing respiratory rate, were all associated with death. The optimal threshold for all indices as well as the CRP, ferritin and LDH was determined using the ROC area under the curve.

**FIGURE 1:**
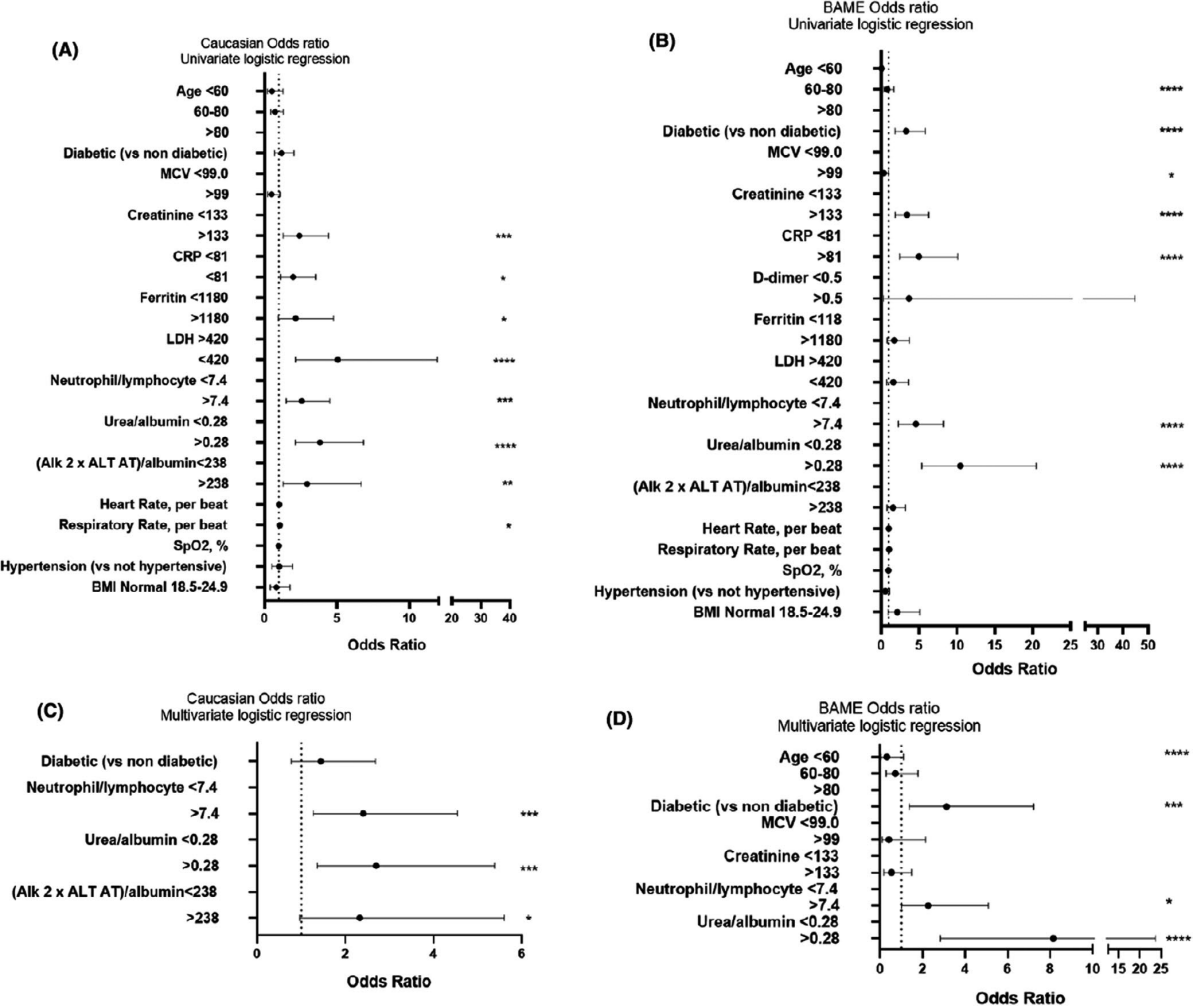
Risk factors associated with in‐hospital death. Univariate risk factors associated with mortality for the Caucasian (plot A) and (plot B) BAME patient groups followed by a multivariate analysis for the Caucasian (plot C) and (plot D) BAME patient groups for 201 and 185 patients with complete data for all variables, respectively. Neutrophil/lymphocyte, urea/albumin, LDH/albumin, CRP/albumin, ferritin/albumin, (ferritin × CRP)/albumin, (Alk 2 × ALT AT)/albumin indices were converted to binary variables according to the optimal cut‐off value by employing receiver operating characteristic (ROC) analysis. OR, odds ratio and statistical significance shown with asterisks where *P ≤ .05, **P ≤ .01, ***P ≤ .001 and ****P ≤ .0001

In the multivariable logistic regression model, we included 201 patients with complete data for all variables (Figure 1). Neutrophil/lymphocyte ratio, and higher urea/albumin ratio and higher (ALP × ALT)/albumin values above 7.4, 0.28 and 238, respectively, increased the odds of death. In the univariable logistic regression analysis for the BAME group, the odds of in‐hospital death were higher in those >60 years and diabetics. Increasing HbA_1_c raised MCV, creatinine, CRP as well as neutrophil/lymphocyte, urea/albumin and (ALP × ALT)/albumin indices above those set by the ROC analysis also increased the risk. We included 185 patients with complete data for all variables in the multivariable logistic regression model (Figure 1). Being older than 60 years of age, diabetic as well as a neutrophil/lymphocyte and urea/albumin ratio above 7.4 and 0.28, respectively, increased odds of death.

## 4 DISCUSSION

This study aimed to analyse differences in blood parameters between the Caucasian and BAME groups of COVID‐19 patients and to further understand risk factors for mortality from a single centre in the West Midlands, UK, with a multi‐ethnic population observing a disproportionally higher death rate.^8^ The study identified several risk factors including new laboratory indices for mortality in both Caucasian and BAME groups. A raised neutrophil/lymphocyte ratio and/or raised urea/albumin ratio were found to be accurate and practical indicators for risk of COVID‐19 associated mortality in both Caucasian and BAME groups. Additionally, among the clinical and demographic parameters in this study, we found age >60 years and diabetes mellitus directly predicted mortality in the BAME group. Further, a raised (ALP × ALT)/albumin ratio was a predictor of COVID‐19 associated mortality in Caucasians only.

Patients with severe COVID‐19 have previously been observed to have a high leucocyte count, low lymphocyte counts and high neutrophil/lymphocyte ratio, as well as lower percentages of monocytes, eosinophils and basophils.^2^, ^5^, ^9^, ^10^, ^16^, ^17^ Lymphopenia and increased neutrophil‐to‐lymphocyte ratio (NLR) (>7.8) in our study was found to be associated with increased mortality, indicating a serious pathological and biochemical disturbance and potential critical condition in severely infected cases^16^ which may be a result of excessive inflammation and immune suppression in sepsis triggered by SARS‐CoV‐2 infection. NLR did not, however, discriminate BAME from Caucasian group perhaps as BAME groups have a lower basal neutrophil count (ethnic neutropenia due to the Duffy gene polymorphism^18^), whereas lymphopenia was worse in the BAME groups suggesting their severity of infection was high. In sepsis, neutrophils are hyper‐activated with delayed apoptosis disorder^19^ a common event in severe COVID‐19.^20^ Recent studies have shown neutrophilia and neutrophil activation signature is present on first day of hospitalization with SARS‐CoV‐2 infection who later on require transfer to intensive care unit. Strongest predictors of critical illness were high resistin, lipocalin‐2, hepatocyte growth factor, interleukin‐8 and granulocyte colony stimulating factor.^21^ Numerous factors may contribute to COVID‐19 associated lymphopenia. Lymphocytes express the angiotensin converting enzyme‐2 (ACE2) receptor on their surface^22^; thus, SARS‐CoV‐2 may directly infect those cells and ultimately lead to their destruction. Whether people within the BAME grouping express excessive ACE2 receptors on their lymphocytes remains a postulation. Furthermore, T‐cell apoptosis,^23^ as well as the cytokine storm, and tumour necrosis factor (TNF)‐alpha, may also promote lymphocyte apoptosis.^4^ Lymphopenia was worse in the BAME groups suggesting their severity of infection was high.

Our results are consistent with those of Bannaga et al,^11^ who showed lower serum albumin in nonsurvivors admitted with COVID‐19. Hypoalbuminaemia may occur as a result of acute inflammatory response and hepatic injury. The raised serum urea concentrations observed could either suggest underlying chronic kidney disease, potentially linked to diabetes, hypertension or acute illness, with dehydration leading to reduced renal perfusion. This was supported by the demonstration of hypernatremia in this study. Hence, attention should be paid in maintaining kidney perfusion and hydration to reduce mortality, particularly in those with chronic kidney disease. Particular attention should be paid to patients admitted to intensive care units, where inotrope support may reduce renal perfusion. The renal dysfunction could also be due to vasculopathy often seen in COVID‐19 patients.^25^

Although we did not observe significant elevations in bilirubin or aspartate aminotransferase (ALT) levels, the multivariate logistic regression analysis observed the (ALP × ALT)/albumin index (>238) was associated with increased odds of death in the Caucasian fatality group. This is likely to be due to hypoalbuminaemia due to tissue leak consequent upon inflammation and perhaps reduced hepatic synthesis.

Older age has been reported as an important independent predictor of mortality.^7^, ^10^ We found age >60 years increased the odds of death within the BAME group. The age‐dependent defects in T‐cell and B‐cell function as well as the excess production of type 2 cytokines could lead to a deficiency in control of viral replication and a more prolonged pro‐inflammatory responses resulting in a poor outcome.^26^ It is, however, not clear why only the BAME population have higher mortality at younger age. Data collected in UK have shown that the BAME population was 1.7‐3.5 times more likely to die from COVID‐19 at any age as compared to Caucasian populations suggesting an either an inherent risk or due to other comorbidities and socioeconomic factors.^27^

Patients diagnosed with diabetes have been reported to have a higher risk of mortality with COVID‐19 infection^28^, ^29^, ^30^. A third of COVID‐19‐related deaths in hospital in England between 1 March 1 and 11 May 2020 were in people with diabetes.^31^ We found diabetes to increase the odds of death in BAME group which may be explained by the higher HbA_1c_ levels in the BAME group. Diabetes is a chronic, low‐grade inflammatory disease often presenting a dysregulated immune response. Furthermore, viral infection may cause fluctuations in blood glucose in diabetic patients thus adversely affecting recovery.^31^ The higher HbA_1c_ concentrations in the BAME group indicate a poor diabetic management of our study population implying poor health care and education of this ethnic group.

Our study has limitations. First, due to the retrospective study design, not all laboratory tests were carried out or recorded in all patients, particularly INR, PTT, D‐dimer, fibrinogen, ferritin, LDH and troponin. Therefore, their role might be underestimated in predicting in‐hospital death. D‐dimer is particularly important as higher levels have been correlated with death.^32^ Secondly, lack of information regarding drug treatment like corticosteroids and immunomodulatory agents might have also influenced the clinical outcomes in some patients. Furthermore, data regarding comorbidities were not intended to be collected in this study; however, data from hospital statistics show that overall 56% of patients had diabetes, 37% had hypertension, 24% had chronic kidney disease and 16% had cancer which could have affected clinical outcomes. Third, interpretation of our findings might be limited by the sample size. However, by including all adult patients across the trust, we believe our study population is representative of cases diagnosed and treated in West Birmingham.

To the best of our knowledge, this is one of the few retrospective studies among patients with COVID‐19 that have compared between the laboratory abnormalities across different ethnic groups. We believe it will be useful for clinicians to use neutrophil/lymphocyte ratio above 7.4 and urea/albumin ratio above 0.28, (ALP × ALT)/albumin index above 238 being (specific to the Caucasian group ) as indicators of severity and consider appropriate intervention to reduce mortality in COVID‐19. Recent evidence of neutrophil activation markers predicting severity of critical illness could confer therapeutic intervention. Furthermore, the BAME subgroup of relatively younger age (>60 years) and those with diabetes are at higher risk of death. They should be shielded or appropriately protected and also intensely monitored should they present with COVID‐19 infection. Attention must be given to good health education about diabetic control in all ethnic groups but more so in BAME groups. With the availability of so many COVID‐19 vaccines, these high‐risk groups should be targeted early for vaccination.

## CONFLICT OF INTEREST

The authors have no competing interests.

## AUTHOR CONTRIBUTIONS

FW, MM and SS designed the study. FW and SS collected the data. MM analysed the data. FW, MM and SS drafted the manuscript. AB, HM and PB critically revised the manuscript. All authors gave final approval for the version to be published.

## ETHICAL APPROVAL

This study was granted ethical approval by the Integrated Research Approval System (289571) and sponsored by research and development committee of the Trust site (20Haem60).

## Supporting information

Table S1‐2
