# Supplementary material for: Analysis of laboratory blood parameter results for patients diagnosed with COVID‐19, from all ethnic group populations: A single centre study
Source: Int J Lab Hematol. 2021 May 3;43(5):1243–51. doi: 10.1111/ijlh.13538 (PMC8239882; doi:10.1111/ijlh.13538)
Supplement: Supplementary file 1 — Table S1‐2 [file IJLH-43-1243-s001.docx]

Supplementary Table 1: Interaction between ethnicity and morbidity on the dependent variable as determined using a general linear model with adjustment for age.

|  | Mortality |  | Ethnicity |  |
| --- | --- | --- | --- | --- |
|  | F* | p value | F | p value |
|  | 10·638 | 0·001 | 2·437 | 0·119 |
| Hb (men) | 1·822 | 0·178 | 5·639 | 0·018 |
| Hb (women) | 5·344 | 0·022 | 2·588 | 0·109 |
| MCV | 8·108 | 0·005 | 40·86 | <0·0001 |
| Platelet count | 0·458 | 0·499 | 0·243 | 0·622 |
| Neutrophil | 14·418 | <0·0001 | 2·381 | 0·124 |
| Lymphocyte | 1·203 | 0·273 | 0·312 | 0·577 |
| Monocyte | 0·008 | 0·931 | 7·267 | 0·007 |
| Eosinophil | 1·236 | 0·267 | 0·994 | 0·319 |
| INR | 0·461 | 0·498 | 1·935 | 0·165 |
| PTT | 3·807 | 0·06 | 0·031 | 0·862 |
| D-dimer | 4·215 | 0·045 | 0·035 | 0·852 |
| Fibrinogen | 5·54 | 0·024 | 5·524 | 0·025 |
| Urea | 21·272 | <0·0001 | 1·787 | 0·182 |
| Sodium | 11·229 | 0·001 | 1·424 | 0·233 |
| Potassium | 5·965 | 0·015 | 9·981 | 0·002 |
| Albumin | 33·627 | <0·0001 | 0·077 | 0·781 |
| Bilirubin | 1·799 | 0·181 | 0·09 | 0·765 |
| ALP | 2·249 | 0·135 | 1·617 | 0·204 |
| ALT | 7·863 | 0·005 | 1·747 | 0·187 |
| Creatinine | 8·275 | 0·004 | 9·07 | 0·003 |
| CRP | 31·5 | <0·0001 | 2·424 | 0·12 |
| Ferritin | 4·665 | 0·032 | 4·158 | 0·043 |
| LDH | 9·942 | 0·002 | 5·244 | 0·023 |
| Troponin | 0·009 | 0·922 | 3·349 | 0·068 |
| HbA_1c_ | 7·363 | 0·007 | 17·375 | <0·0001 |
| Neutrophil/lymphocyte | 18·34 | <0·0001 | 1·58 | 0·209 |
| Urea/albumin | 7·85 | 0·005 | 13·59 | <0·0001 |

*F: Factor

Supplementary Table 2: Non-normally and normally distributed continuous variables for the black and Asian population are presented as medians (interquartile ranges, IQR) and mean (standard deviation) respectively as well as n/N (%). Continuous variables were analysed using a Mann-Whitney U test.

|  | Survivors |  |  | Mortality |  |  |
| --- | --- | --- | --- | --- | --- | --- |
|  | Black | Asian | p value | Black | Asian | p value |
| WBC, × 10^9^ /l | 8.30 [6.3-10.8]  (n = 21) | 7.50 [5.80-9.40]  (n = 53) | 0.29 | 8.35 [6.75-10.65]  (n = 32) | 8.60 [6.85-11.78]  (n = 36) | 0.91 |
| Hb (men) g/l | 144 [115.5-152.8]  (n = 9) | 135 [114-143.5]  (n = 30) | 0.33 | 121 [102-134]  (n = 19) | 117 [105.5-137.3]  (n = 24) | 0.73 |
| Hb (women) g/l | 124 [111-127]  (n = 12) | 117 [105.5-127.5]  (n = 23) | 0.61 | 111 [90.5-199.5]  (n = 13) | 109 [83.5-134]  (n = 12) | 0.88 |
| MCV, × 10^9^ /l | 85.30 [81.00-91.40]  (n = 21) | 84.20 [78.90-88.60]  (n = 53) | 0.70 | 88.95 [82.93-91.45]  (n = 32) | 89.10 [83.70-93.45]  (n = 36) | 0.66 |
| Platelet count, × 10^9^ /l | 243.0 [207.0-308.0]  (n = 21) | 23.00 [177.00-325.00]  (n = 51) | 0.64 | 226.0 [175.5-282.8]  (n = 32) | 223.5 [166.8-275.0]  (n = 36) | 0.75 |
| Neutrophil , × 10^9^ /l | 6.59 [4.92-9.16]  (n = 21) | 5.33 [4.07-7.97]  (n = 53) | 0.15 | 6.75 [5.63-9.57]  (n = 32) | 5.32 [6.96-10.39]  (n = 36) | 0.91 |
| Lymphocyte , × 10^9^ /l | 1.19 [0.79-1.55]  (n = 21) | 1.14 [0.79-1.54]  (n = 51) | 0.21 | 0.81 [0.59-1.40]  (n = 32) | 0.71 [0.51-1.11]  (n = 36) | 0.24 |
| Monocyte , × 10^9^ /l | 0.5 [0.39-0.81]  (n = 21) | 0.45 [0.29-0.70]  (n = 53) | 0.38 | 0.38 [0.25-0.67]  (n = 32) | 0.48 [0.27-0.58]  (n = 36) | 0.88 |
| Eosinophil , × 10^9^ /l | 0.005 [0.005-0.02]  (n = 21) | 0.01 [0.005-0.09]  (n = 51) | 0.08 | 0.008 [0.005-0.108]  (n = 32) | 0.01 [0.005-0.04]  (n = 36) | 0.85 |
| Basophil, × 10^9^ /l | 0.02 [0.01-0.03]  (n = 21) | 0.02 [0.01-0.04]  (n = 53) | 0.77 | 0.02 [0.01-0.04]  (n = 32) | 0.02 [0.01-0.03]  (n = 36) | 0.72 |
| INR | 1.16[1.08-1.22]  (n = 21) | 1.14 [1.06-1.30]  (n = 38) | 0.79 | 1.19 [1.12-1.33]  (n = 23) | 1.19 [1.11-1.46]  (n = 29) | 0.93 |
| PTT | 1.03  (n = 1) | 0.88 [0.87-1.00]  (n = 3) | ·· | 1.01 [0.92-3.35]  (n = 3) | 1.04 [0.91-1.31]  (n = 5) | 0.73 |
| D-dimer µgFEU/m | 0.22  (n = 1) | 0.22  (n = 1) | ·· | 128  (n = 1) |  |  |
| Fibrinogen g/l | 10  (n = 1) | 5.20 [3.00-8.9]  (n = 3) | .·· | 10 [9.00-10.00]  (n = 7) | 6.95 [3.83-8.65]  (n = 8) | 0.03 |
| Urea mmol/l | 8.1 [4.86-15.38]  (n = 20) | 6.20 [4.15-10.15]  (n = 52) | 0.17 | 12.60 [8.20-22.50]  (n = 31) | 13.90 [8.85-19.83]  (n = 38) | 0.78 |
| Sodium mmol/l | 138.50 [134.80-142.00]  (n = 20) | 135.00 [132.00-137.30]  (n = 52) | 0.01 | 141.0 [136.0-143.0]  (n = 31) | 138.0 [134.0-143.0]  (n = 38) | 0.37 |
| Potassium mmol/l | 4.25 [3.53-4..50]  (n = 16) | 4.30 [3.75-4.65]  (n = 47) | 0.49 | 4.60 [3.78-5.3]  (n = 30) | 4.15 [3.73-4.68]  (n = 36) | 0.18 |
| Albumin g/l | 35.00 [33.00-39.00]  (n = 17) | 37.00 [33.00-41.00]  (n = 52) | 0.75 | 32.00 [26.75-39.00]  (n = 30) | 33.50 [25.25-37.75]  (n = 36) | 0.90 |
| Bilirubin µmol |  | 5  (n = 1) | ·· | 13  (n = 1) |  |  |
| ALP U/l | 101.0 [75.00-142.0]  (n = 17) | 76.00 [65.00-107.50]  (n = 47) | 0.10 | 84.5 [58.75-107.3]  (n = 30) | 89.00 [60.00-105.0]  (n = 35) | 0.90 |
| ALT U/l | 38.00 [26.00-63.00]  (n = 17) | 28.50 [20.00-45.75]  (n = 46) | 0.20 | 35.00 [18.75-58.75]  (n = 30) | 35.00 [18.00-59.00]  (n = 35) | 0.78 |
| Creatinine µmol/l | 100.5 [86.25-162.0]  (n = 20) | 92.50 [71.50-127.80]  (n = 52) | 0.30 | 132.0 [72.00-299.0]  (n = 31) | 129.5 [88.75-207.5]  (n = 38) | 0.57 |
| CRP mg/l | 152.00[40.5-218.5]  (n = 18) | 99.00 [43.00-185.0]  (n = 45) | 0.09 | 187.0 [115.3-275.5]  (n = 28) | 149.0 [96.00-232.3]  (n = 38) | 0.34 |
| Ferritin µg/l | 1329 [141.0-2440]  (n = 11) | 289.5 [172.8-925.0]  (n = 26) | 0.83 | 860.5 [206.5-3464]  (n = 14) | 495.0 [184.0-1742]  (n = 23) | 0.59 |
| LDH U/l | 444.5 [354.3-625.0]  (n = 8) | 337.5 [284.5-477.3]  (n = 22) | 0.10 | 682.0 [521.0-846.0]  (n = 11) | 406.0 [253.5-625.8]  (n = 18) | 0.006 |
| Troponin ng/l | 18.50 [7.75-63.25]  (n = 10) | 6.00 [3.50-45.25]  (n = 28) | 0.15 | 140.0 [60.50-353.0]  (n = 17) | 46.00 [22.00-166.0]  (n = 27) | 0.04 |
| HbA_1c_ mmol/mol | 42.00 [39.00-53.00]  (n = 17) | 43.00 [40.00-61.00]  (n = 47) | 0.34 | 52.00 [45.00-63.00]  (n = 31) | 52.00 [44.00-67.5]  (n = 37) | 0.90 |

*in some instances the number of patients with available data was very small or zero, as a results the values were not calculated (··)
